# Structural analysis of urate oxidase in complex with its natural substrate inhibited by cyanide: Mechanistic implications

**DOI:** 10.1186/1472-6807-8-32

**Published:** 2008-07-20

**Authors:** Laure Gabison, Thierry Prangé, Nathalie Colloc'h, Mohamed El Hajji, Bertrand Castro, Mohamed Chiadmi

**Affiliations:** 1 LCRB, UMR CNRS 8015, Université Paris Descartes, 4 avenue de l'Observatoire, 75270 Paris cedex 06, France; 2 CI-NAPS, UMR 6232, UCBN, CNRS, centre CYCERON, Boulevard Becquerel, 14074 Caen cedex, France; 3 SANOFI-AVENTIS Recherche & Développement, Rue du Pr Blayac, 34184. Montpellier, France

## Abstract

**Background:**

Urate oxidase (EC 1.7.3.3 or UOX) catalyzes the conversion of uric acid and gaseous molecular oxygen to 5-hydroxyisourate and hydrogen peroxide, in the absence of cofactor or particular metal cation. The functional enzyme is a homo-tetramer with four active sites located at dimeric interfaces.

**Results:**

The catalytic mechanism was investigated through a ternary complex formed between the enzyme, uric acid, and cyanide that stabilizes an intermediate state of the reaction. When uric acid is replaced by a competitive inhibitor, no complex with cyanide is formed.

**Conclusion:**

The X-ray structure of this compulsory ternary complex led to a number of mechanistic evidences that support a sequential mechanism in which the two reagents, dioxygen and a water molecule, process through a common site located 3.3 Å above the mean plane of the ligand. This site is built by the side chains of Asn 254, and Thr 57, two conserved residues belonging to two different subunits of the homo-tetramer. The absence of a ternary complex between the enzyme, a competitive inhibitor, and cyanide suggests that cyanide inhibits the hydroxylation step of the reaction, after the initial formation of a hydroperoxyde type intermediate.

## Background

Urate oxidase (uricase; EC 1.7.3.3; or UOX) belongs to the purine degradation pathway and catalyzes in the presence of molecular oxygen the hydroxylation of uric acid into a metastable product identified as the 5-hydroxyisourate (5-HIU) [1]. Once released in solution, 5-HIU decays slowly to allantoin, a process independent of oxygen and associated with the release of CO_2 _(dehydro-decarboxylation).). *In vivo*, 5-HIU is rapidly processed by two specific enzymes to [S]-allantoin [2,3], the structures of which were solved [4,5]. Urate oxidase is present in many species, but is absent in human and higher apes. This emphasizes an evolutionary advantage since it was suggested that uric acid being a powerful anti-oxidant, humans would have less free radicals, so less cancer due to aging. As a consequence, uric acid level in plasma is quite elevated and a higher pathological level may be fatal. Sanofi-Aventis produces and commercializes urate oxidase, first extracted from *Aspergillus flavus *and now expressed in *Saccharomyces cerevisiae*, branded under the name Fasturtec^® ^(DCI : Rasburicase), to prevent hyperuricemia that can happen during chemotherapies of children.

The *Aspergillus flavus *urate oxidase crystallizes in the orthorhombic system, space group I222. The asymmetric unit contains one monomer of 301 amino-acids and the whole tetramer is built using the two-fold axes of the current I222 crystal symmetry. The complete structure has the shape of a barrel 70 Å high, with an inner and outer radius of about 6 Å and 30 Å, respectively [6]. Each monomer is associated with one active site located at a dimer interface. The four active sites are accessible from the external surface of the tetramer. The role and significance of the central void channel still remains unknown.

The catalytic mechanism of urate oxidase is original since it does not imply any cofactor or metal ion, questioning about how urate, a singlet, can react with oxygen, a triplet. Several X-ray structures with, or without uric acid analogues have already been determined to unravel the three-dimensional active site topology [7,8]. Grown in presence of uric acid (the natural substrate), the crystalline functional enzyme readily degrades its substrate and catches back (*Kd *~10^-7^) the final product of the reaction cascade, the S-allantoin [9]. Under dioxygen pressure, and in the presence of the competitive inhibitor 8-azaxanthine (8-AZA), the location of molecular oxygen in the active site was recently characterized giving information about the first step of the reaction [10]. Here, in the presence of sodium cyanide, known to compete with dioxygen [11], we crystallize a non productive ternary [UOX/uric acid/cyanide] complex, that shows for the first time the natural substrate within the active site of UOX. In the same structure, a cyanide ion is observed at the location occupied by molecular oxygen in the first step of the mechanism [10] or by a water molecule in the following hydroxylation step of the reaction [7,8]. All attempts to crystallize a ternary [UOX/8-AZA/cyanide] complex prevent any cyanide anion to be observed in the crystal structure.

## Results

**Figure 1:**
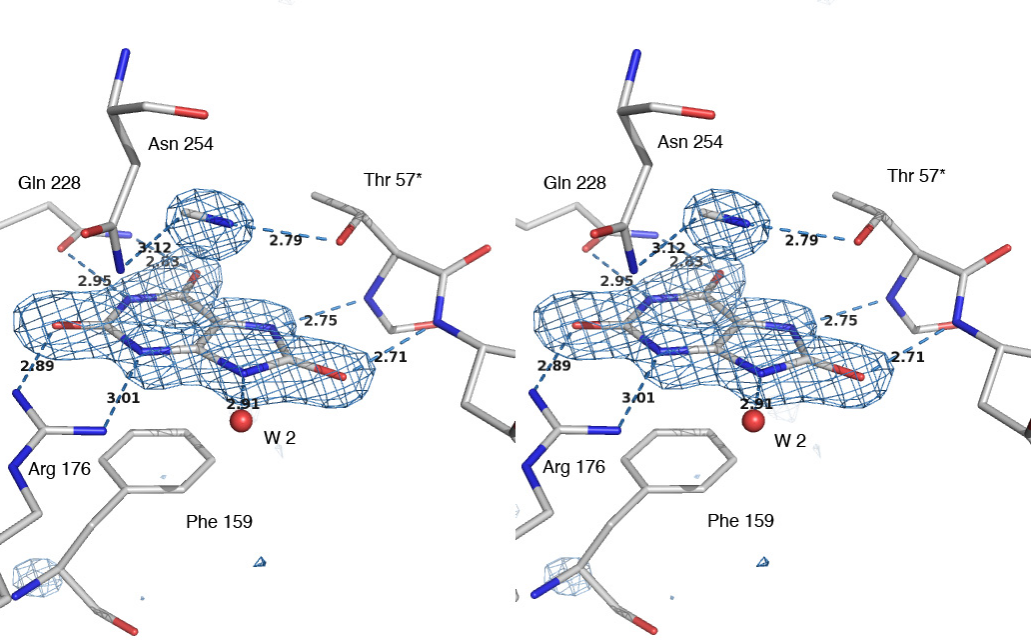
**Omit-map showing the electron density corresponding to the two partners of the [UOX/UA/CN] complex, the cyanide and uric acid.** The active site is delimited by 1) the conserved residues implicated in the molecular tweezers (Arg 176, Glu 228) that holds the substrate, 2) the Phe 159 closing one end of the cavity below, and 3) the two Asn 254 and Thr 57* forming another tweezers above the mean plane of the ligand that construct a location where efficient electron transfer can take place at a low energy level via the catalytic triad Thr 57* Lys 10* His 256, and the associated water molecules shown in Figure 3). The orientation of cyanide is based on a coherent evolution of the C/N thermal factors during refinements, compared to neighboring atoms (stereo view).

In normal reactive conditions, UOX crystals grown in the presence of uric acid lead to a complex between the protein and the final product of degradation, S-allantoin showing the high affinity of UOX for the last product of the reaction cascade [9]. The electron density map of the ternary [UOX/UA/CN] crystal structure shows a density clearly corresponding to the natural substrate that was never observed before because of its rapid degradation by the enzyme. The elongated density observed at stacking distance (3.3 Å) of the mean-plane of urate, and attributed to the expected cyanide anion, fills a site where either a dioxygen or a catalytic water molecule have been previously observed [10] – Figure 1. This represents so far the first observation of a non heme-complexed cyanide ion in a crystalline structure. On the contrary, in the complex with the 8-AZA inhibitor, no equivalent signature of cyanide could be observed and the perfect spherical density at the cyanide location indicates that the catalytic water is still present with its centroid corresponding to the nitrogen atom of the cyanide. We then conclude that the substrate and the cyanide ion are both necessary for the formation of a stable and non-productive intermediate state.

Compared to the structure of a previously solved non-cyanide 8-AZA complex [UOX/8-AZA] (*2iba *from PDB), both complexes, the ternary [UOX/UA/CN] and the [UOX/8-AZA] crystallized in cyanide, show very little deviation on coordinates with an average r.m.s.d of 0.19 Å over all the main chain atoms. However, an important feature distinguishes complexes crystallized with cyanide from the others. At pH above 9, that invariably occurs upon addition of a cyanide excess, a flip of the His 98 is observed with a χ_1 _angle moving from -60° to 180°, leading to a complete rearrangement of the hydrogen bond network in this region. In all previously solved structures at pH below (or close to) 9, the imidazole group of His 98 was observed as a positively charged group because it is hydrogen bonded on both sides to carboxylates of Asp 100 and Glu 131. Increasing the pH above 9 by cyanide addition favors an uncharged His 98 at the origin of this drastic rearrangement.

The different structures already solved with urate-like inhibitors have shown that the ligand is stacked with phenylalanine 159 and always hydrogen-bonded to the enzyme through a molecular tweezers (the "ligand tweezers") composed by the two invariant residues arginine 176 and glutamine 228 [7,8]. In [UOX/UA/CN], the urate substrate (Figure 1) is exactly positioned as observed in all other inhibited UOX structures (this includes 8-azaxanthine, xanthine, 8-nitroxanthine, 9-methyl uric acid, oxonic acid and di-aminouracil). On top of the ligand, at a distance of 3.3 Å, the catalytic water molecule W1, usually hydrogen bonded by the "reagent tweezers" built by the side-chains of asparagine 254 and threonine 57* from a symmetric subunit [7-9], is now replaced by a cyanide anion. When the "reagent tweezers" holds a single water molecule, the distance between the Oγ and Nδ2 atoms of the tweezers is remarkably constant (5.53 ± 0.05 Å) as shown in structures *1r4u*, *1wrr*, *1xxj*, *1xt4*, and *2iba *from the PDB. In the [UOX/8-AZA] structure crystallized in cyanide, this distance falls within these limits (5.48 Å), an additional argument defending the lack of cyanide in the electron density. In the case of the ternary [UOX/UA/CN] complex, the cyanide insertion now significantly increases this distance up to 5.89 Å, close to what is observed in the oxygen-pressurized [UOX/O_2_/8-AZA] structures *2zka*, *2zkb *or *3cks *[10], a feature underlining the flexibility of this motif. When the reagent tweezers is void of any ligand, like in *1r56 *or *1xy3 *structures, the distance increase up to 6 Å.

**Figure 2:**
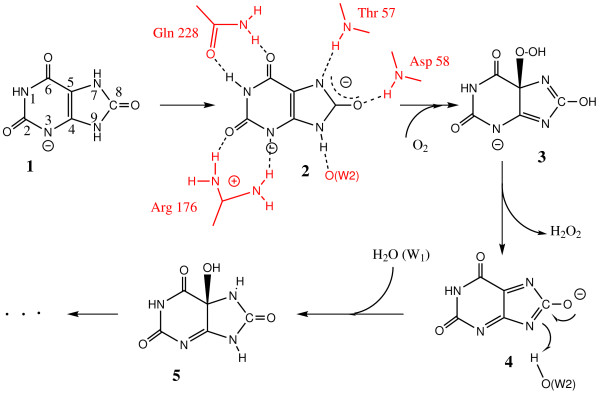
**The reaction pathway from compiled data**[10,18,19]. Although the use of Lewis-type representation is inadequate for strongly mesomeric structures (radicals or anions), this sketches about the different intermediates **1**to **4**involved in the mechanism leading to 5-HIU, **5**.

**Figure 3:**
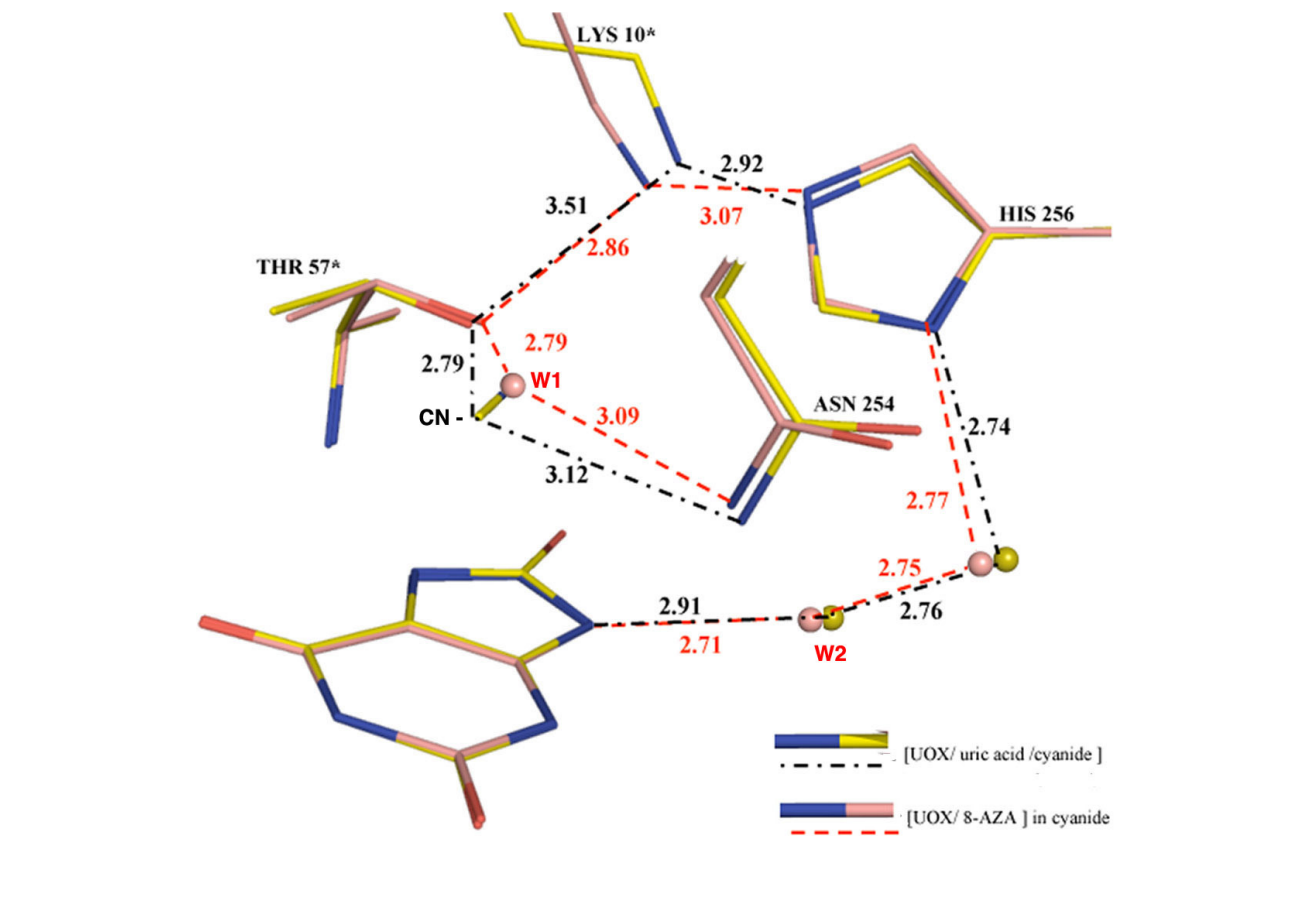
**The superimposition of the catalytic triad TKH in the two structures [UOX/UA/CN] and [UOX/8-AZA] crystallized in cyanide.** This shows how the cyanide present in the first structure influences the H-bond network (black dotted lines for cyanide/uric acid and pink dotted lines for 8-AZA complexes). The permanent negative charge of the cyanide induces a drastic change in the H-bond network continuity with a nearly broken Thr 57* – Lys 10* connection (3.51 Å compared to 2.86 Å in the non-cyanide 8-AZA complex). A similar discontinuity is observed when a chloride anion is located in the oxygen/W1 hole [10].

In addition, W1 and the Oγ of Thr 57* (building the peroxo hole) are the starting point of a complex proton relay including Lys 10*, His 256, and two water molecules, ending to W2 and to the N9 atom of the ligand, as shown in Figure 2 and 3. However, Thr 57* in [UOX/UA/CN], cannot act as a base because of the charged cyanide ion. The threonine 57* becomes a proton donor instead and as a result, the extended electron relay through the already mentioned Lys 10* and His 256 residues is interrupted as shown by the increased distance of 3.5 Å between Lys 10* and Thr 57* (Figure 3).

All the ligands so far observed in the peroxo hole (water, oxygen, cyanide, chloride ..) are always tightly bound as illustrated by low thermal B factors, full occupancy factors – Table 1) and well defined electron densities, including the Lys 10* side-chain.

**Table 1:** Crystallographic data collection and refinement statistics. The two structures are orthorhombic, space group I222.

**Complex structure**	**[UOX/UA/CN]**	**[UOX/8-AZA/CN]**
Unit cell parameters (Å)		
a =	79.94	79.72
b =	94.99	95.10
c =	104.29	103.96
Resolution range (Å) *	30 – 1.8 (1.9 – 1.8)	30 – 1.6 (1.7 – 1.6)
No of measured reflections*	147 946 (20 420)	255 591 (36 780)
No of unique reflections*	35 679 (5250)	51 972 (7545)
R_merge _(%) overall *	6.4 (22.6)	4.4 (10.4)
Completeness (%) *	96.6 (98.4)	99.5 (100)
Redundancy *	4.1 (3.9)	4.9 (4.9)

**Refinements:**		

R_work _(%)^#^	21.8	20.0
R_free _(%)^¥^	24.0	22.2
No of waters	234	279
Average thermal factors (Å^2^)		
Main chain atoms:	16.6	14.7
Side-chain atoms:	21.2	18.7
Water molecules:	25.2	26.3
Ligands	(CN-): 15.5 (Urate): 13.4	(W1): 10.6 (8-AZA): 11.2
Rms Deviations:		
Bond distances (Å)	0.006	0.005
Bond angles (°)	1.3	1.4
Planes (Å)	0.21	0.19
Chiral Volumes (Å^3^)	0.030	0.035
PDB access number	3BJP	3BK8

* Last shell values are given in parentheses.

^# ^The crystallographic R-factor is defined as ∑|*F*_*o*_| - |*F*_*c*_|/∑|*F*_*o*_|

^¥^Calculated using 8 percent of the native data, which were randomly chosen and excluded from refinements

## Discussion

As mentioned above, the catalytic mechanism of urate oxidase is of particular interest because UOX is a cofactor-less enzyme that requires no special assistance [12]. Isotopic labeling experiments have proved that the oxygen atoms of hydrogen peroxide derived from dioxygen and that the oxygen atom attached to C5 in the product derived from a water molecule [13]. NMR and spectroscopic studies have shown that UOX transforms the urate anion in a metastable compound identified as 5-hydroxyisourate (5-HIU) [1,14-16] and not allantoin. In addition, it was suggested from stop-flow kinetics experiments that urate hydro-peroxide would be a primary intermediate, presumably through the addition of a superoxide radical O2^-. ^[16]. Although this intermediate was not firmly established, the structural similarities between UOX, pterin and other reduced flavin proteins reinforces this suggestion [17].

Following a number of observations and spectroscopic results, a multi-step mechanism, summarized in Figure 2, was proposed [10,18,19]. In this model, stepwise additions of dioxygen and a water molecule occur sequentially *via *the same catalytic site (the peroxo hole). Further, a recent EPR investigation of the reaction [20] demonstrated that intermediates are essentially radicals and not ionic species as indicated in Figure 2. As imperfect as this model can be, it represents up to now the best hypothesis for this particular mechanism.

### Mono-urate, di-urate or dehydrourate anions in the active site?

Two distinct enzymatic intermediates were proposed in the initial steps of the urate oxidase reaction, the urate dianion and 5-hydroperoxyisourate (5-HPIU), based on their spectral signatures [16] – Scheme 1. At the end of the first step, the elimination of hydrogen peroxide from 5-HPIU would yield to dehydrourate that is not experimentally observed [18], probably because it does not accumulate during the enzymatic turnover. The final step of the reaction is the rapid nucleophilic addition of a hydroxyl group deriving from water W1 onto dehydrourate to give 5-HIU and a re-protonation by the water molecule W2 (Figures 1 and 3). The characterization of discrete intermediates during the urate oxidase reaction ruled out any concerted mechanism in which W1 would attack at C5 simultaneously with direct electron transfer from urate to oxygen.

Since the urate pk's are 5.3 and 10.3, the mono N3-deprotonated form of the substrate is likely to be the species which first binds at biological pH. At high pH, non-enzymatic solution studies (voltametric) have shown a more facile oxidation of the dianion form of urate than the monoanion [21]. The formation of urate dianion after binding was evidenced and proposed to result from the predicted action of an unprotonated residue involved in a base catalysis [12,22]. Quantum mechanic calculations are in agreement of the [N3^- ^N7^-^] dianion as it displays the lowest ionization potential once complexed in the active site and as such becomes the most eligible to give spontaneously one of its electrons to an acceptor species [23].

Mutation of critical residues [18] suggested that a threonine involved in the active site may be a good candidate to act as the expected general base in the deprotonation. The X-ray structure suggests that the proton transfer to the solvent by a lysine [18] is in relay with an histidine [10] and assisted by several water molecules. This complex relay, which initiates at the catalytic water molecule W1 and terminates at W2 the water molecule that re-protonates the N9 atom of the ligand (Figure 3), is thus able to play the role of a "proton shuttle" in the reaction.

Obviously, the X-ray structure of this ternary complex represents a static view of an intermediate state of the reaction and question arises about the exact ionic state of the bound ligand observed in the active site (Figure 1). A survey of the protonation state at proximity of the ligand, although hydrogen atoms cannot be directly observed in the electron density, indicates that two of them are not present as deduced from the donor/acceptor state of the side chains around it. According to this, it is likely that the nearly flat electron density observed in the active site would not correspond to mono-urate but can be either the urate dianion [N3^- ^N7^-^] (intermediate 2 in Figure 2), the radical mono anion, or the dehydrourate mono anion (intermediate 4 in Figure 2).

### Inhibition by cyanide

It is known that UOX is inhibited in solution by cyanide with a loss of activity of 90 % [11,24,25]. The same holds for azide, but at a lesser extent [25], questioning at that time about the presence of a heavy metal in the active site, as many metallo-oxidases are analogously poisoned by cyanide. For example, irreversible inhibition is observed when xanthine oxidase is incubated with cyanide in the absence of substrate. By analogy, cyanide was assumed to interact with the dioxygen site in UOX, although not involved in the formation of any intermediate structure [26]. However, compared to xanthine oxidase, the inhibition of UOX by cyanide is non competitive *versus *urate, increases with time, and is perfectly reversible [11].

The location of the cyanide anion, which replaces either the molecular dioxygen involved in the hydroperoxy intermediate formation, or the water molecule (W1) involved in the hydroxylation of the dehydrourate intermediate, suggests that the cyanide inhibits any access to the peroxo hole during the course of the reaction. When urate, ^14^C-labeled cyanide, and dioxygen are present together in the incubation mixture, a labelled enzyme complex is detected [11]. Removing one of the three components from the incubation mixture, or replacing either O_2 _by N_2_, did not show the formation of a labeled complex. Due to a packing effect, this experiment would be difficult to interpret in the crystalline state, because of additional labelings at interfaces. We have undertaken NMR experiments in solution using ^13^C cyanide to confirm these results. A clear signal corresponding to a bound ion to the protein is observed in presence of oxygen and uric acid. No labeling is observed when urate is replaced by 8-AZA, an observation that may explain why no cyanide is observed in the [UOX/8-AZA/CN] structure. Moreover, the initial velocity of urate transformation in the absence of cyanide did not change after extensive pre-incubation of the enzyme with cyanide [11]. These results suggest more precisely the generation of a cyanide inhibitor site in the simultaneous presence of the two substrates, urate and O_2_. Thus, cyanide presumably reacts once an intermediate already oxidized by O_2 _is formed. Moreover, the presence of a dianion and a charged cyanide, that represent three negative charges in the same closed environment, would be an energetically non favorable situation. In these conditions, it is likely that the flat electron density observed in the active site (Figure 1) would best correspond to the second stage of the reaction, *i.e*. the complexed dehydrourate intermediate rather than the urate dianion (Figure 2). This supports that cyanide would mainly compete reversibly with water in the second step of the reaction rather than oxygen in the first step.

## Conclusion

Two non-productive ternary complexes of urate oxidase that mimic two different stages of the reaction are now available: the first structure [UOX/8AZA/O2], with 8-azaxanthine, an analogue of uric acid, and oxygen recently determined [10] and the present structure [UOX/UA/CN^-^], which includes the natural substrate and cyanide, a competing inhibitor of oxygen. This allows us to characterize the two reaction partners within the active site and derive additional information about the mechanism.

First, we confirm that in all cases, the enzyme holds either uric acid or its analogues in the same orientation within the active site. Second, the oxygen site previously characterized [10] is occupied by a cyanide ion only when the natural substrate is present, suggesting that the binding of uric acid must precede in normal conditions the binding of dioxygen. As such, these two UOX complexes describe two different intermediate steps of the reaction pathway and suggest a common catalytic site where both di-oxygen and water (and probably hydrogen peroxide) are sequentially driven. This mechanism shares important similarities with other cofactor-less oxidases [27], and also with catalase, the enzyme that performs the reverse reaction of hydrogen peroxide to dioxygen, also known to sequentially drive hydrogen peroxide and water molecules through a common catalytic site [28].

## Methods

Purified recombinant urate oxidase (UOX) from *A. flavus*, expressed in *S. cerevisiae*, was supplied by Sanofi-Aventis Co. The crystals of the ternary [UOX/uric acid/cyanide] complex [UOX/UA/CN] were grown following the batch technique at room temperature, using conditions slightly modified from [29]. 5–10 mg/ml protein was dissolved in 50 mM Tris/HCl pH 8.5 and NaCN 0.2 M. To the solution, 10–15% w/v PEG 8000 was added. After a rapid mixing, orthorhombic crystals started growing within a few hours. They were rapidly harvested, before they develop to full size and immediately transferred in a cryoprotectant mixture of the same crystallizing solution plus 20 % v/v of 2,4-dimethyl pentane diol (MPD), or ethylene glycol for one minute, then flash-cooled in liquid nitrogen. The same process was followed and the same crystalline form obtained when replacing uric acid by its analogue 8-azaxanthine [UOX/8-AZA/CN] in the crystallization medium.

X-ray data collections were carried out at the ESRF (Grenoble, France) BM14 beam line, at a wavelength of 0.978 Å, operating in the 16 bunch mode, using a MAR CCD detector. The temperature was set to 100 K. Data were integrated by the *MOSFLM *program [30], amplitudes were derived after merging and scaling using the *SCALA *and *TRUNCATE *programs [31]. The starting model for rigid body refinement was taken from the refined UOX in complex with 8-nitro-xanthine [UOX/8-NXN], the coordinates of which were refined at a near atomic resolution (Gabison *et al*., in preparation) after removing the water molecules and the ligand. Structure refinement was carried out by *CNS *[32]. The graphics program *O *[33] was used to visualize |*2Fobs - Fcalc*|, |*Fobs - Fcalc*|, and omit electron-density maps and for manual refittings.

### [UOX/UA/CN] complex

Following two steps of positional and thermal parameters refinement, an electron density map was calculated showing a density corresponding clearly to the natural substrate (Figure 1). In addition, the elongated electron density, about 3.3 Å near the mean plane of urate was attributed to the expected cyanide anion, at a location where a water molecule is always present in other inhibited UOX structures. The ligand coordinates determined by semi-empirical calculations [34] were then included in the structure, as well as the cyanide. The complete model was improved by subsequent refinement cycles followed by manual re-fitting of some residues in the electron density, including Arg 7, His 98, and Gln 131 side chains. Particular attention was taken about the solvent molecules by examining their interactions and the spherical shape of their electron density.

### [UOX/8-AZA] cyanide complex

Equivalent protocol for refinements was adopted, using the *CNS *program in a similar way. The 8-AZA coordinates were taken from a previously equivalent structure (2IBA, Protein Data Bank). However, no signature of the cyanide ion was visible in subsequent improved electron difference maps.

The two coordinate data sets were deposited with the Protein Data Bank (ref. number 3BJP and 3BK8). As evaluated by *PROCHECK *[35], all non-glycine residues fall within the energetically favorable regions of a Ramachandran plot [36]. The figures were prepared using *PYMOL *[37]. Summaries of the data collection statistics and refinements for the two structures are shown in table 1.

## Authors' contributions

LG, TP, and MC carried out the crystallizations, the X-ray data recordings and analyses, MEH and BC manufactured and provided the urate oxidase, TP, MC, and NC designed the study and wrote the paper. All authors read and approved the final manuscript.
